# Supplementary material for: In Situ Monitoring of Aptamer–Protein Binding on a ZnO Surface Using Spectroscopic Ellipsometry
Source: Sensors (Basel). 2023 Jul 13;23(14):6353. doi: 10.3390/s23146353 (PMC10385375; doi:10.3390/s23146353)
Supplement: Supplementary file 1 [file sensors-23-06353-s001.zip › sensors-2476535-supplementary.pdf]

Supplementary Materials

In Situ Monitoring of Aptamer–Protein Binding on a ZnO Surface Using Spectroscopic Ellipsometry

Adeem Alshammari, Harm van Zalinge and Ian Sandall *

Department of Electrical Engineering & Electronics, University of Liverpool, Liverpool L69 3GJ, UK; vzalinge@liverpool.ac.uk (H.v.Z.)
* Correspondence: isandall@liverpool.ac.uk

S1

(a)

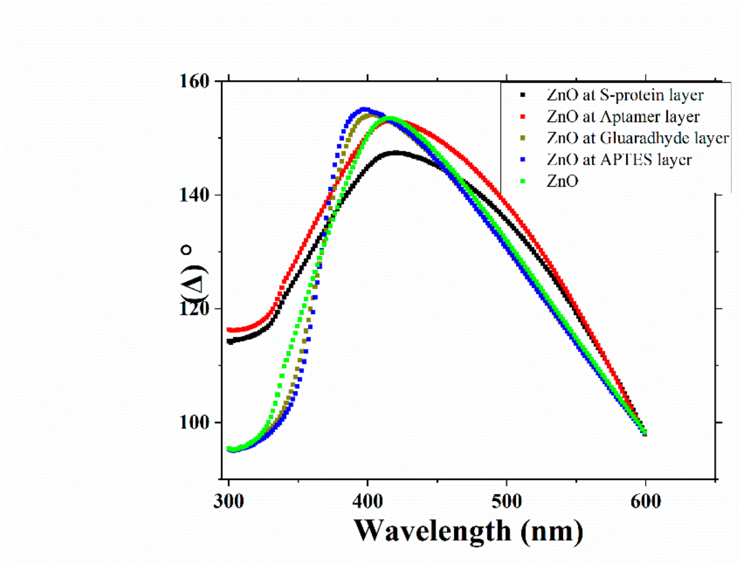

(b)

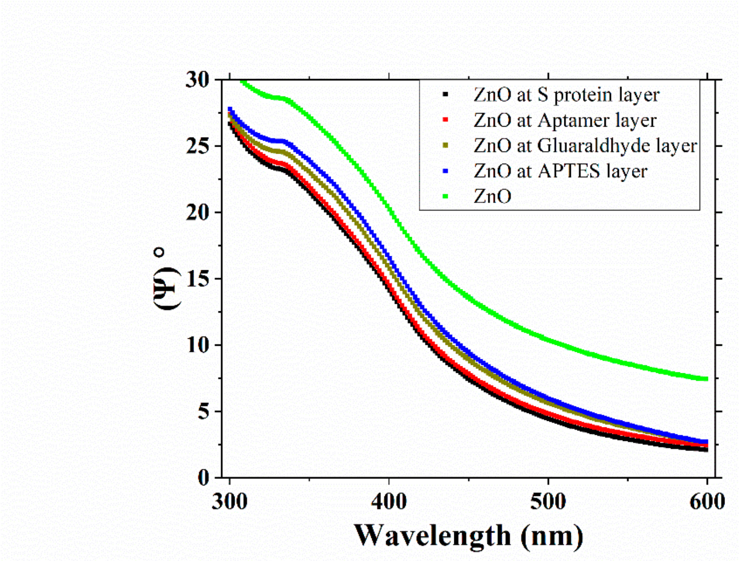

Figure S1. Ellipsometeric paramaters (a) $\Delta(\lambda)$*and (b)* $\Psi(\lambda)$ for the ZnO thin film at several stages of fabrication: bare ZnO, after APTES silanisition , after glutargenization, with aptamer attached, and with 125nM of spike protein.

A clear shift is observed in both the Psi and Delta spectra onces the APTES layer is attached to the ZnO. Upon each further step smaller further changes in the spectra are also noted. As a rule the change in

shape is more pronunced in the delta spectra with the psi values showing moe of a rigid shift after each step, but maintaining the same general shape.

S2

From the spectra shown in S1 the Refractive index and thickness for each layer are determined by fitting a Cauchy model for each layer. These values have an error associated with them, the table below shows the values and errors extracted from the model for each spectrum for the in situ measurments taken for the 125 Nm Spike protien concentration measurment. These errors are then included in the De Feijter equation using standard error analysis. A similar percentage error was obtained for all the layers investigated in this work.

Table S1 – Measured errors in Refractive index and thickness for spectra taken every 2 minutes for in-situ measurments with 125 nM spike protien exposure.

Time (min)	RI	Er±	Thickness (nm)	Er±
1	1.334	0.0024	0	0.001
3	1.3338	0.0024	0	0.001
5	1.3342	0.089	0	0.005
7	1.333	0.25	0	0.001
9	1.3343	0.24	0	0.002
11	1.3987	0.066	8.22	0.001
13	1.4018	0.45	3.72	0.038
15	1.41625	0.011	7.41	0.001
17	1.4042	0.029	2.85	0.03
19	1.46951	0.048	7.44	0.001
21	1.55241	0.013	9.62	0.001
23	1.55022	0.0078	9.89	0.012
25	1.49767	0.027	9.9	0.007
27	1.49855	0.036	9.72	0.003
29	1.4989	0.026	9.72	0.002
31	1.5239	0.066	9.62	0.007
33	1.49135	0.023	4.24	0.001
35	1.48961	0.06	4.19	0.002
37	1.48351	0.04	4.16	0.001
39	1.47308	0.09	4.16	0.001
41	1.47308	0.036	4.15	0.002
43	1.47518	0.065	4.12	0.001
45	1.47463	0.058	4.11	0.003
47	1.49825	0.066	4.13	0.001
49	1.49766	0.012	4.13	0.002
